# Manifestations of Boron-Alkali Metal and Boron-Alkaline-Earth Metal Romances

**DOI:** 10.1021/acs.accounts.5c00852

**Published:** 2026-02-18

**Authors:** Zhong-hua Cui, Li-juan Cui, Jorge Barroso, Jin-Chang Guo, Hua-jin Zhai, Sudip Pan, Gabriel Merino

**Affiliations:** † Institute of Atomic and Molecular Physics, Jilin University, Changchun 130023, China; ‡ State Key Laboratory of Inorganic Synthesis and Preparative Chemistry, 528579 Jilin University, Changchun 130023, China; § Department of Chemistry, 428597 Clemson University, Clemson, South Carolina 29634, United States; ∥ Nanocluster Laboratory, Institute of Molecular Science, Shanxi University, Taiyuan 030006, China; ⊥ Departamento de Física Aplicada, 130299 Centro de Investigación y de Estudios Avanzados, Unidad Mérida. Km 6 Antigua Carretera a Progreso. Apdo. Postal 73, Cordemex, 97310 Mérida, Yuc., México

## Abstract

The electron deficiency of boron promotes the formation of multicenter σ and π bonds that endow its clusters and solids with exceptional structural diversity. While bulk boron favors cage-like frameworks, clusters often adopt planar or quasi-planar motifs composed of triangles that evolve into tubular and cage-like architectures as their size increases. Many of these clusters are stabilized by delocalized σ and π bonds that are associated with fluxional behavior and multiple aromaticity.

Metal doping enriches this chemistry. Transition metals use their *d* or *f* orbitals to couple with the boron framework, generating metal-centered rings, metallo-boron nanotubes, and metalloborophenes. In contrast, alkali and alkaline-earth metals have long been viewed as simple counterions, yet recent findings reveal that they can orchestrate deep structural reorganizations by combining charge transfer with efficient orbital overlap. Lithium, for example, leads to a quasi-planar → tubular → cage evolution in B_12_ clusters via strong electrostatic attraction to the boron framework, whereas beryllium engages in pronounced covalent Be–B interactions that yield rare architectures such as the Archimedean Be_4_B_12_^+^ cage, the B–Be sandwich B_7_Be_6_B_7_, and four-ring tubular forms like Be_2_B_24_^+^.

In heavier alkaline-earth systems, the participation of (n–1)*d* orbitals (Ca, Sr, Ba) introduces transition-metal-like covalent interactions, producing highly symmetric rings and tubular clusters. This Account summarizes how electrostatic and covalent interactions jointly control geometry and bonding in boron–metal systems, defining the rich landscape of boron chemistry.

## Graphical abstract

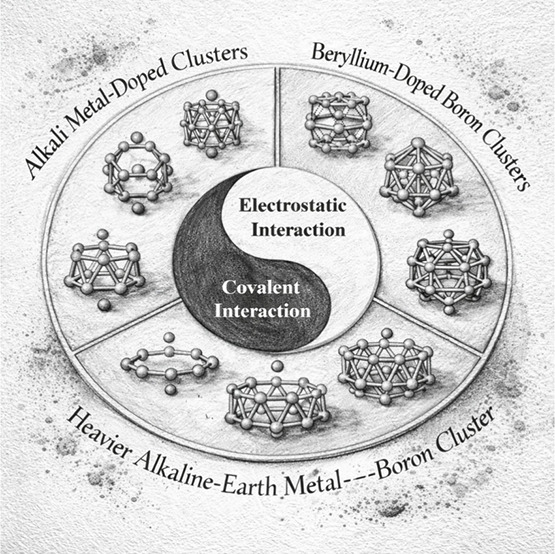

## Key References

- Dong, X.; Tiznado, W.; Liu, Y. Q.; Leyva-Parra, L.; Liu, X. B.; Pan, S.; Merino, G.; Cui, Z. H. B_7_Be_6_B_7_: A Boron–Beryllium Sandwich Complex. Angew. Chem. Int. Ed. 2023, 62, e202304997 10.1002/anie.20230499737268596.^1^
- This study reports the first global minimum of a boron-based sandwich complex. A structure with a central Be_6_ ring coupling two flanking borozene units. The combination of electrostatic and covalent interactions is shown to account for the exceptional thermochemical and kinetic stability of the B_7_Be_6_B_7_ motif.
- Dong, X.; Liu, Y. Q.; Liu, X. B.; Pan, S.; Cui, Z. H.; Merino, G. Be_4_B_12_^+^: A Covalently Bonded Archimedean Beryllo-Borospherene. Angew. Chem. Int. Ed. 2022, 61, e202208152 10.1002/anie.20220815236028732.^2^
- Here, it is shown that beryllium doping promotes a three-dimensional cage in small boron clusters. In particular, Be_4_B_12_^+^ adopts a truncated-tetrahedral geometry that can be described as an Archimedean beryllo-borospherene, highlighting the role of covalent Be–B bonding beyond purely electrostatic effects.
- Dong, X.; Jalife, S.; Vásquez-Espinal, A.; Ravell, E.; Pan, S.; Cabellos, J. L.; Liang, W. Y.; Cui, Z. H.; Merino, G. Li_2_B_12_ and Li_3_B_12_: Prediction of the Smallest Tubular and Cage-Like Boron Structures. Angew. Chem. Int. Ed. 2018, 57, 4627–4631 10.1002/anie.20180097629473272.^3^
- In this work, lithium doping is shown to drive a planar-to-tubular-to-cage structural evolution in the B_12_ framework. Strong electrostatic interactions between Li^+^ cations and the boron core overcompensate the distortion penalty of the quasi-planar structure, enabling the formation of the smallest tubular and cage-like boron motifs in Li_2_B_12_ and Li_3_B_12_.
- Cui, L. J.; Dong, X.; Liu, Y. Q.; Pan, S.; Cui, Z. H. Transition Metal Behavior of Heavier Alkaline Earth Elements in Doped Monocyclic and Tubular Boron Clusters. Inorg. Chem. 2024, 63, 653–660 38146259 10.1021/acs.inorgchem.3c03536.^4^
- This study shows that heavier alkaline-earth metals (Ca, Sr, and Ba) exhibit transition-metal-like behavior in doped boron clusters. Covalent interactions involving the (n–1)*d* orbitals of these metals are identified as the key stabilizing factor for monocyclic and multiring tubular boron frameworks.

## 1 Introduction

The electron deficiency of boron leads to multicenter bonding patterns that support a variety of architectures, from two-dimensional (2D) sheets^5–8^ to three-dimensional (3D) cages.^9–11^ Because boron has only three valence electrons, it forms delocalized σ and π bonding networks by extensively sharing electrons.^12,13^ This leads to structural richness of boranes and bulk boron allotropes, particularly those composed of B_12_ icosahedra.^14–17^

In contrast to bulk boron, boron clusters display distinct structural preferences. Smaller clusters often adopt planar or quasi-planar geometries built from triangular units,^18–20^ a motif that persists in anionic species such as B_40_^–^.^10^ As cluster size increases, the 2D forms gradually evolve into tubular (B_20_),^21^ cage-like borospherenes (B_39_^–^),^9^ and fullerene-like (B_40_)^10^ structures. This preference for planarity originates from boron’s electron deficiency and its ability to form multicenter bonds.^22,23^ Midsized clusters such as B_30_^–^ and B_33_^–^ to B_38_^–^ display planar arrangements with mixed polygonal voids,^24–28^ bridging clusters and extended boron sheets (borophenes) synthesized on metallic and inert surfaces.^29,30^

Electron deficiency is therefore responsible for not only structural diversity but also distinct electronic properties, including σ- and π-double aromaticity and high symmetry.^31^ These features yield closed-shell clusters such as B_7_^3–^, B_8_^2–^, and B_9_^–^ that parallel the aromatic hydrocarbons C_5_H_5_^–^, C_6_H_6_, and C_7_H_7_^+^, respectively.^32–34^ The analogy between boron clusters and organic aromatic systems shows how delocalized multicenter bonding can replicate π-aromatic stability in a purely inorganic setting.^22,32^

Beyond their structural diversity, boron clusters are also known to exhibit fluxionality, a dynamical property rooted in their electron-deficient nature and the prevalence of multicenter σ and π bonding.^35^ When bonding is sufficiently delocalized and not constrained by localized two-center interactions, multiple equivalent structures may interconvert through low-energy pathways, leading to large-amplitude nuclear motions even at low temperatures. Canonical examples include B_19_^–^ and B_13_^+^,^36–39^ in which an inner polygon undergoes rotational motion within an outer ring, leading to interconversion among equivalent geometries through low-barrier rearrangements. Such behavior has been documented in both pure and metal-doped boron clusters.^35^

Doping with transition metals further expands this landscape.^40–44^ Metal incorporation enables new topologies and bonding patterns.^45–48^ For example, species such as M©B_n_^–^ retain planarity despite high coordination numbers, reaching ten in Ta©B_10_^–^ and Nb©B_10_^–^.^47^ Larger systems form metal-centered boron drums such as MB_16_^–^ (M = Co, Mn),^49,50^ and metalloborophenes MB_18_^–^ (M = Rh, Co),^51,52^ where coupling between metal *d* orbitals and boron π networks determines both stability and dimensionality.

Transition-metal doping also yields half-sandwich,^53–56^ inverse-sandwich,^57–59^ and cage-like species,^60–65^ showing how the metal valence configuration and the participation of *d* and *f* orbitals shape the bonding framework. Collectively, these examples highlight boron clusters as a unifying platform to examine how multicenter bonding, delocalization, and boron–metal cooperativity define the boundary between classical main-group and transition-metal chemistry.

While electrostatic and covalent contributions coexist and continuously evolve through a balance of charge transfer and orbital participation, alkali and alkaline-earth metals interact with boron clusters mainly through electrostatic interactions, reflecting their tendency to donate their valence electrons, in contrast to transition metals.^1–3,66–75^ Studies of such doped systems have shown that multiply charged boron frameworks can function as robust ligands and structural building blocks.^42,43^

Lithium induces pronounced structural changes in boron clusters. Its strong electrostatic attraction with the boron frameworks drives the conversion of quasi-planar B_12_ clusters into tubular (Li_2_B_12_) and cage-like (Li_3_B_12_) architectures (Scheme 1a).^3^ In larger clusters such as B_24_, the presence of two lithium atoms promotes the formation of three-ring tubular motifs that overcome the energetic cost of framework distortion (Scheme 1b).^73^ In contrast, beryllium, despite also being an *s*-block element, stabilizes rare architectures through covalent Be–B interactions arising from the mixing of its 2*s* and 2*p* orbitals with the delocalized orbitals of the boron framework, while retaining a non-negligible electrostatic component. Representative examples include the Archimedean beryllo-borospherene Be_4_B_12_^+^,^2^ the four-ring tubular beryllo-borospherene Be_2_B_24_^+^,^74^ and the Be–B sandwich B_7_Be_6_B_7_ (Scheme 1c).^1^

**Scheme 1:**
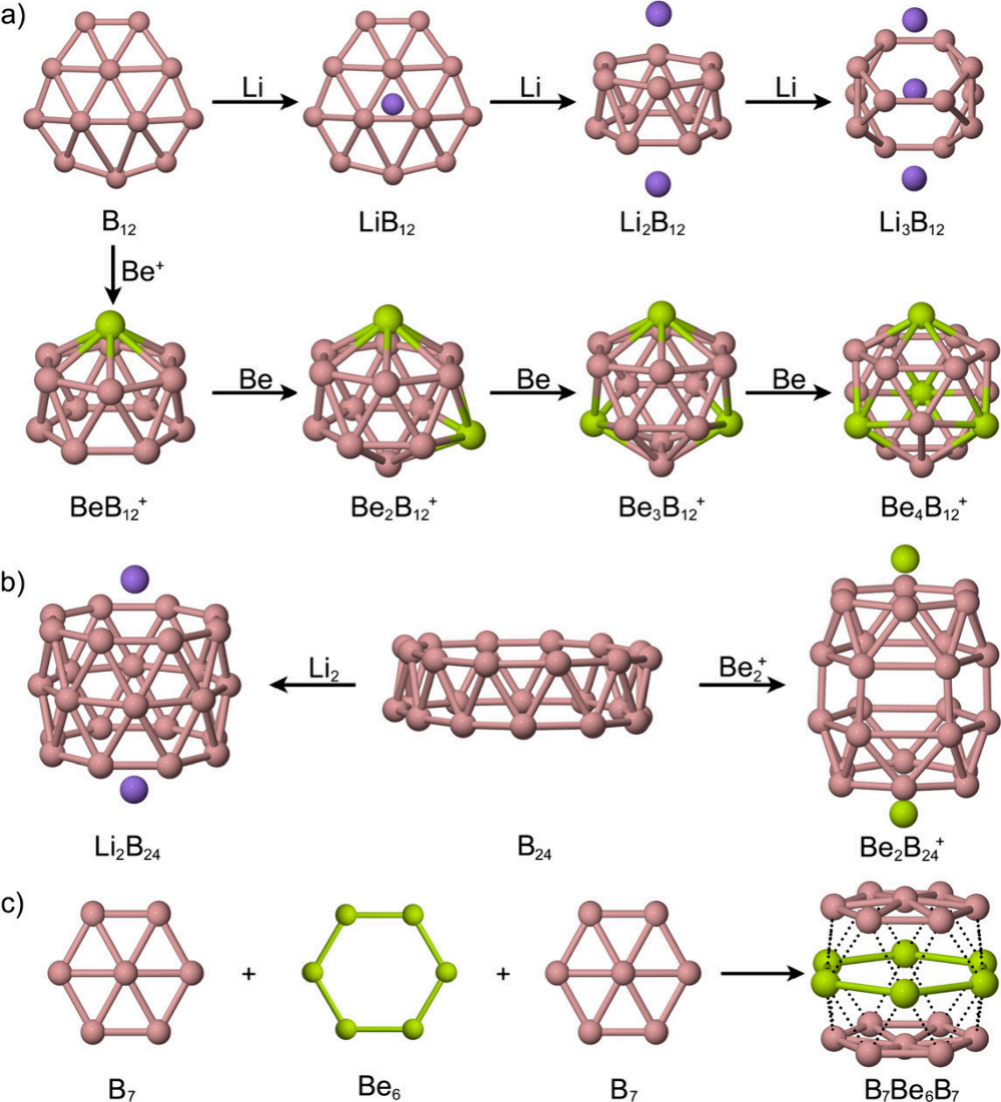
Structural Evolution of Boron Clusters Induced by Alkali and Alkaline-Earth Metals

For heavier alkaline-earth elements (Ca, Sr, Ba), the participation of the (n–1)*d* orbitals introduces a new and distinct bonding regime in boron clusters.^4,76^ This *d*-orbital involvement confers covalent character to the boron–metal interactions, enabling highly symmetric monocyclic and tubular geometries that are reminiscent of transition-metal behavior in contrast to typical main-group bonding.^4^ Together, these findings establish alkali and alkaline-earth metal doping as an effective strategy to modulate geometry, electronic delocalization, and aromaticity in boron clusters, defining the central theme of this Account.

## 2 Electrostatic Interaction between Alkali Metals and Boron Clusters

### 2.1 From Quasi-planar to Tubular and Cage-like Structures in Li_n_B_12_ (n = 1–3)

B_12_ adopts a *C*_3*v*_ quasi-planar geometry stabilized by a delocalized π framework analogous to that of benzene.^77^ Upon reduction to B_12_^–^ and B_12_^2–^, the geometry remains largely unchanged, indicating that the additional electrons have little influence on the boron skeleton.^77,78^ These extra electrons, however, increase Coulombic repulsion within the cluster, suggesting that the introduction of cations may facilitate charge redistribution and promote the stabilization of alternative geometries.

Surprisingly, exploration of the potential energy surfaces (PESs) of Li_n_B_12_ (n = 1–3) reveals a structural evolution upon sequential Li addition.^3^ The lowest-energy LiB_12_ species adopts a half-sandwich geometry, in which the quasi-planar B_12_ ring binds the Li atom (Figure 1), an arrangement reminiscent of transition-metal-doped clusters such as CoB_12_^–^ and RhB_12_^–^.^55^ Upon the introduction of a second Li atom, the framework undergoes a pronounced reorganization. Li_2_B_12_ adopts a *D*_6*d*_-symmetric double-ring tubular structure that lies about 5 kcal/mol below its quasi-planar counterpart. Addition of a third Li atom yields Li_3_B_12_, a *C*_*s*_ cage composed of two B_3_ rings bridged by boron dimers. Slight distortions, mainly associated with the Jahn–Teller effect,^79^ produce short B–B distances in the bridging B_2_ units (<1.60 Å), indicating partial multiple-bond character.^3^

**Figure 1:**
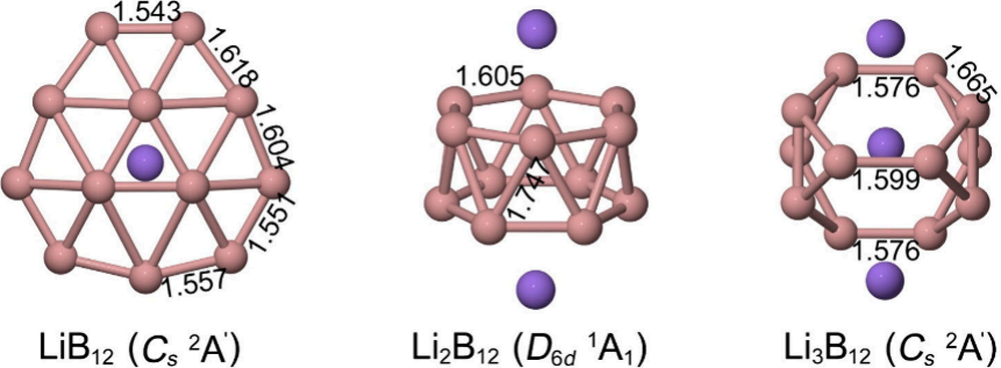
Lowest-energy structures of LiB_12_, Li_2_B_12_, and Li_3_B_12_ clusters.

Population analysis indicates that each Li atom carries a charge close to +1, consistent with strong charge transfer from Li to the boron skeleton. The resulting species can therefore be described as Li_n_^n+^B_12_^n–^ complexes in which electrostatic interactions dominate.

To rationalize the stabilization of tubular and cage-like forms upon Li doping, isomerization energy decomposition analysis (IEDA) was applied.^80,81^ Within this approach, the total isomerization energy is partitioned into distortion and interaction terms, allowing a direct comparison of the physical factors that favor one geometry over another. For Li_2_B_12_ (Figure 2), the tubular structure is stabilized primarily by an increase in electrostatic attraction between the Li_2_^2+^ dimer and the distorted boron framework. Although deformation of the boron skeleton incurs an energetic cost, this penalty is offset by a reduction in Coulombic repulsion between the Li atoms and by stronger Li–B electrostatic interactions, which together account for the energetic preference for the tubular isomer.^3^

**Figure 2:**
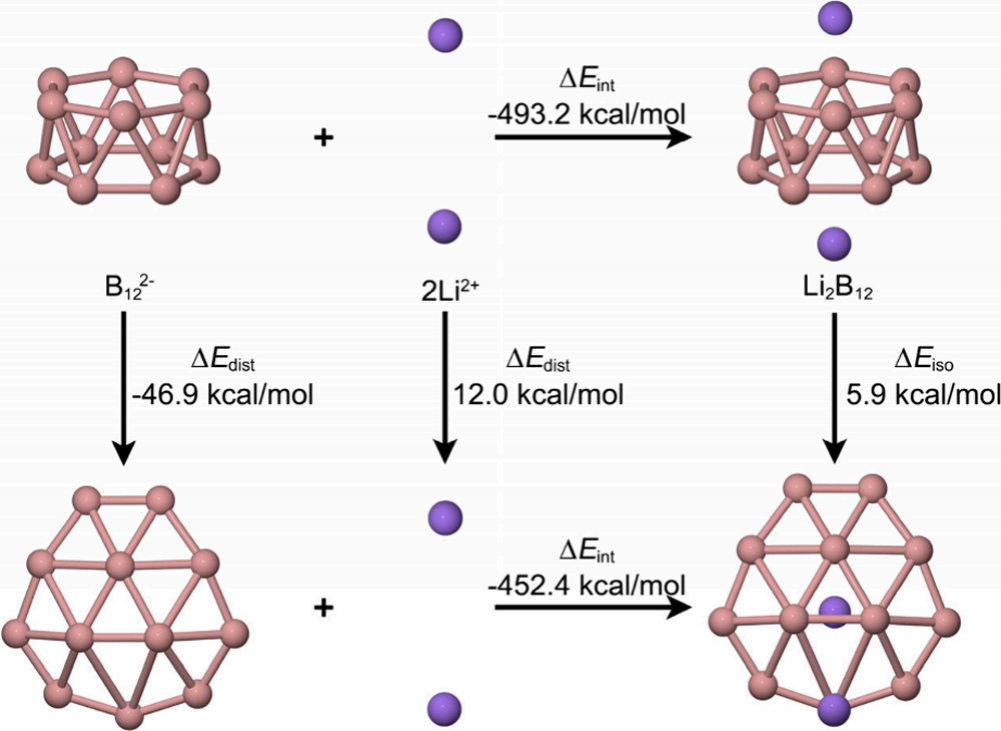
Energetic reaction cycle involving the isomerization of the Li_2_B_12_ between the quasi-planar (bottom) and tubular (top) structures.

To rationalize the bonding patterns discussed below, we briefly outline the Adaptive Natural Density Partitioning (AdNDP) approach.^13^ AdNDP interprets molecular electronic structure in terms of *n*-center two-electron (*n*c–2e) bonds, where *n* ranges from one to the total number of atoms in the system. In this way, AdNDP recovers both classical Lewis bonding elements, such as lone pairs and two-center–two-electron (2c–2e) bonds, as well as multicenter delocalized bonding motifs associated with electron delocalization and aromaticity, without invoking resonance structures. The method is based on analysis of the first-order reduced density matrix in the natural atomic orbital basis and provides a unified description of systems featuring coexisting localized and delocalized bonding, which is particularly well suited for electron-deficient boron and boron–metal clusters.

In the case of LiB_12_, AdNDP shows that the system retains localized (2c–2e) σ bonds along the periphery, whereas Li_2_B_12_ is characterized by three delocalized 14c–2e (σ + σ) and one 14c–2e (σ–σ) bonds satisfying the Hückel rule,^82^ in addition to three delocalized π bonds (Figure 3). These findings confirmed that σ and π aromaticity act cooperatively within the boron framework, stabilizing the tubular and cage-like forms through Li-induced charge donation and the associated reorganization of multicenter delocalization.^3^

**Figure 3:**
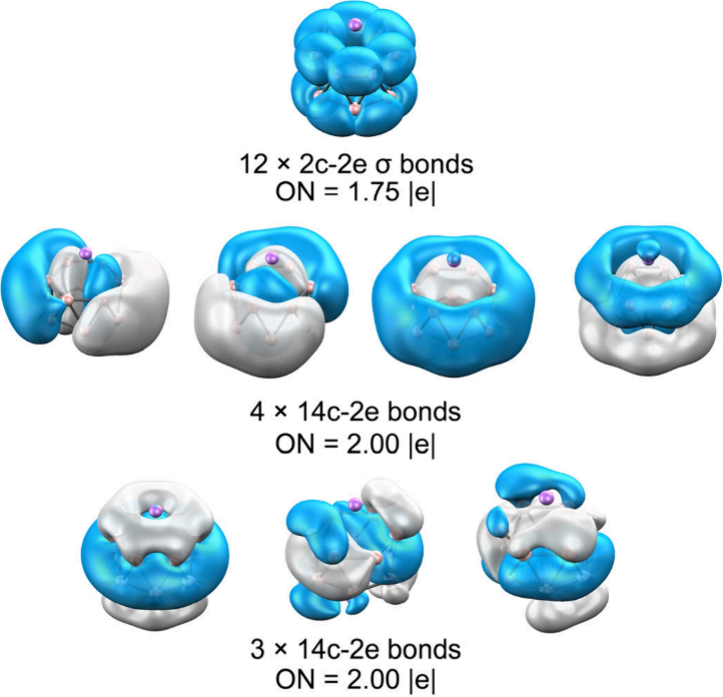
AdNDP bonding analysis of Li_2_B_12_. Occupation numbers (ON) are given in |e|.

### 2.2 The Simplest Three-Ring Boron Tube

B_24_ exhibits a delicate balance between quasi-planar and tubular forms.^83,84^ At the CCSD­(T)/6–311+G­(d)//PBE0/6–311+G­(d) level, the double-ring tubular structure is the most stable isomer, while the planar form lies only slightly higher in energy (13.1 kcal/mol).^73^ Upon addition of electrons, as in B_24_^–^, B_24_^2–^ and B_24_^3–^, the planar motifs become energetically favored, showing how changes in charge shift the balance between 2D and 3D structures.^83–85^ This sensitivity to electron count reflects a characteristic feature of boron chemistry, in which multicenter bonding adapts to electronic perturbations.

Lithium doping shifts this equilibrium toward tubular motifs. The addition of two Li atoms converts B_24_ into a *D*_8*h*_-symmetric three-ring tubular structure, with each Li capping one end of the tube.^73^ This beautiful geometry establishes a direct structural connection to extended boron nanotubes (Figure 4). In this arrangement, the Li atoms reside near the centers of the terminal rings, donating close to one electron each to the boron framework. The resulting charge transfer strengthens electrostatic attraction and favors the formation of a highly delocalized σ–π network across the entire tube.

**Figure 4:**
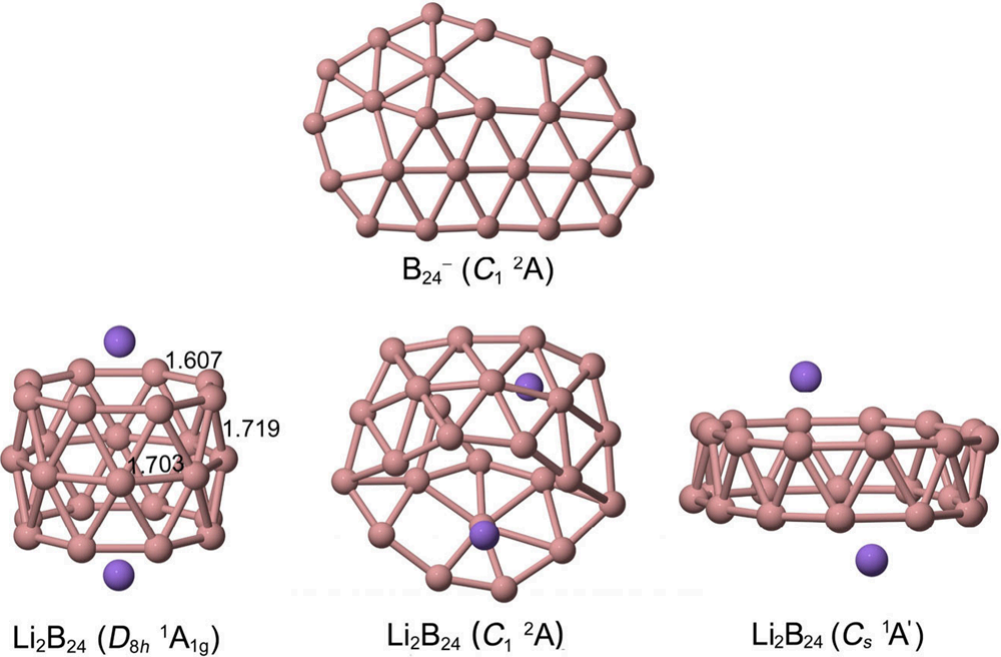
Global minimum of the B_24_^–^ clusters (top), and low-lying Li_2_B_24_ isomers: three-ring tubular (**1A**), amorphous (**1B**), and double-ring (**1F**), shown from left to right.

IEDA confirms that electrostatic interactions constitute the dominant stabilizing contribution, outweighing the energetic cost associated with bending the boron framework. All components of the interaction energy, except Pauli repulsion, favor the tubular form, indicating that Li_2_B_24_ is stabilized primarily by electrostatic charge donation coupled to electronic delocalization within the boron skeleton.

AdNDP further supports this picture (Figure 5). The σ framework comprises peripheral 2c–2e B–B bonds together with multicenter 4c–2e σ bonds connecting adjacent rings. In addition, several sets of delocalized 24c–2e σ and π orbitals extend over the entire structure, each satisfying the Hückel rule. This combination of σ, π, and mixed σ–π delocalization gives rise to triple aromaticity, which, together with electrostatic stabilization from Li^+^ cations, accounts for the high stability and symmetry of the three-ring tubular structure.

**Figure 5:**
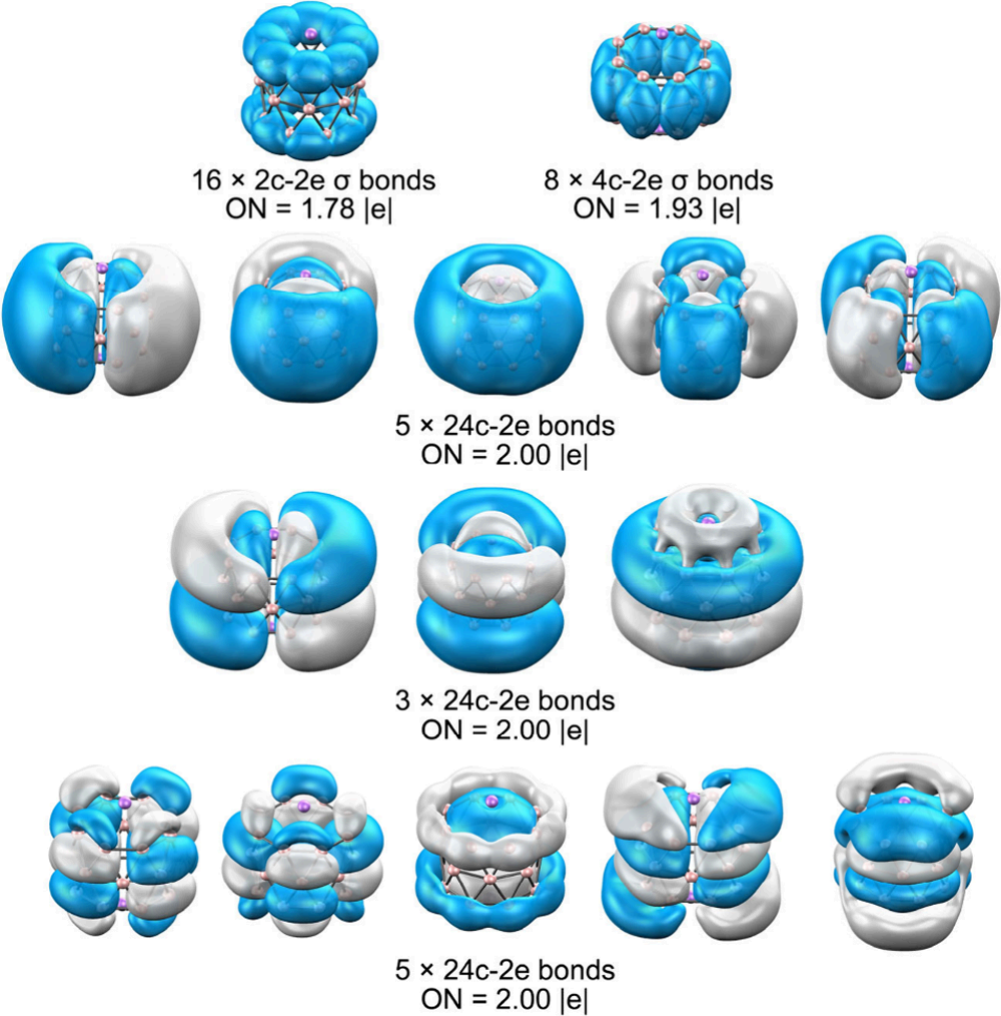
AdNDP analysis of Li_2_B_24_. Occupation numbers (ON) are shown in |e|.

## 3 Strong Electrostatic and Covalent Interactions in Beryllium-Doped Boron Clusters

### 3.1 A Covalently Bonded Beryllo-Borospherene in Be_n_B_12_^+^ (n = 2–4)

In the cationic series Be_n_B_12_^+^ (n = 2–4), the global minima correspond to closed boron cages in which Be atoms occupy hexagonal vacancies, forming the so-called beryllo-borospherenes (Figure 6).^2^ Among them, Be_4_B_12_^+^ adopts a beautiful truncated-tetrahedral cage stabilized by four B_6_ rings, each coordinated to one Be atom. This near-tetrahedral structure (formally *D*_2*d*_ due to the Jahn–Teller distortions) shows the emergence of covalent Be–B interactions arising from mixing between the Be 2*s*/2p orbitals and the delocalized orbitals of the boron framework, in clear contrast to the predominantly electrostatic bonding in lithium-doped clusters.

**Figure 6:**
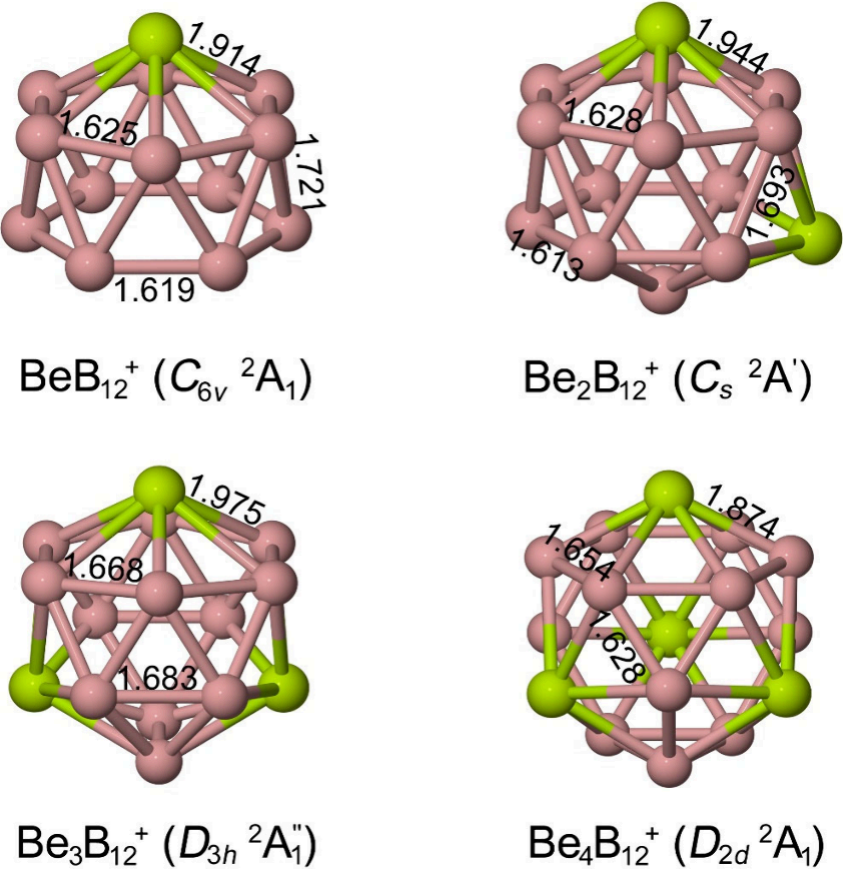
Geometries and selected bond distances of global-minimum isomers of Be_n_B_12_^+^ (*n* = 1–4). Bond distances are in Å.

Bonding analysis based on AdNDP for Be_4_B_12_^+^ (Figure 7) indicates a combination of localized 3c–2e σ bonds within the boron triangles and extended multicenter orbitals delocalized over the entire cage. The Be atoms contribute mainly via their 2*s* and 2*p* orbitals, while natural population analysis shows a partial charge transfer of about +1.7 |e| per Be atom, consistent with strong Be→B_12_ donation that reinforces the covalent skeleton.

**Figure 7:**
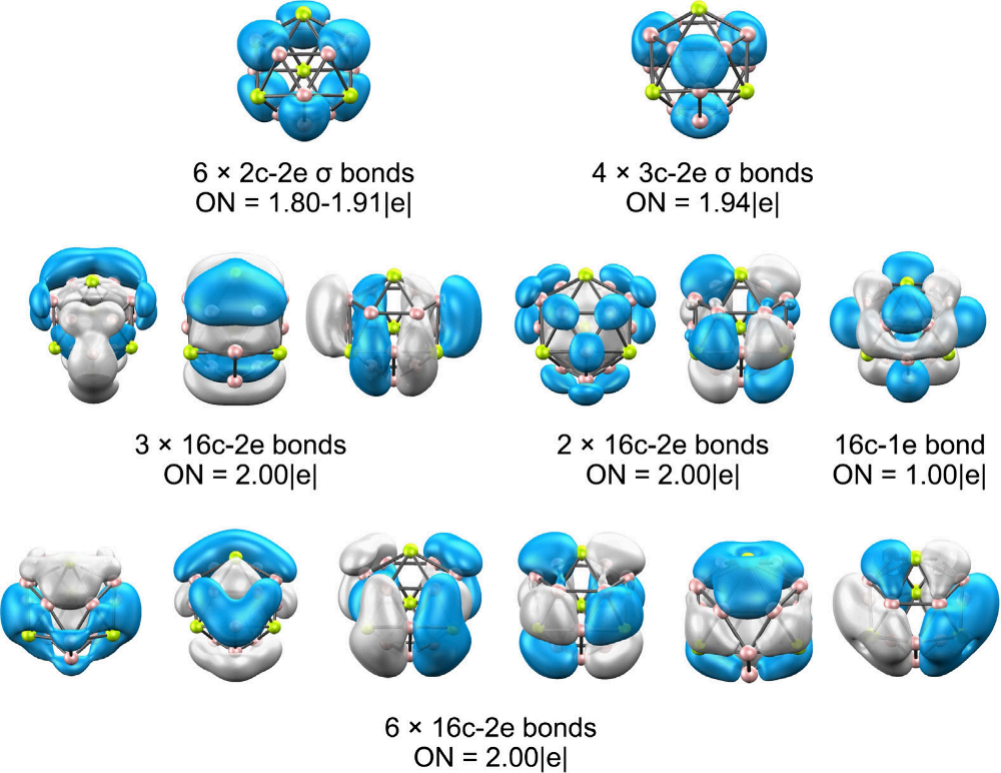
AdNDP analysis of Be_4_B_12_^+^. Occupation numbers (ON) are shown in |e|.

Energy Decomposition Analysis with Natural Orbitals for Chemical Valence (EDA-NOCV)^86–88^ further clarifies the nature of this bonding. The interaction is dominated by polarized electron sharing between Be^+^(2p_∥_) fragments and the Be_3_B_12_ cage, accounting for roughly two-thirds of the total attraction, while the remaining contribution arises from electrostatic components. Accordingly, the bonding can be described as a combination of covalent Be^+^(p_∥_) ↔ B_12_ electron-sharing and Be^+^(p_∥_) ← B_12_ dative interactions. Minor Be 3*d* orbital contributions are also found in the bonding analysis; however, these correspond to polarization and acceptor effects involving essentially unoccupied 3*d* orbitals. This behavior contrasts with that in heavier alkaline-earth elements, where the (*n*-1)*d* orbitals plays a decisive and more covalent role.

### 3.2 A B–Be Sandwich Complex in B_7_Be_6_B_7_

Planar boron clusters are often viewed as inorganic analogues of aromatic hydrocarbons because their delocalized π systems resemble those of classical arenes.^23,32,89^ Yet, unlike organic rings such as cyclopentadienyl or benzene, boron frameworks rarely form sandwich-type complexes, as adjacent units typically fuse through direct B–B bonds rather than stacking via π–π interactions.^8,31^ This contrast raises the question of whether boron frameworks can emulate organometallic sandwich motifs when combined with an appropriate metal layer.

Our systematic exploration of B_n_–Be_m_ combinations (n = 3–14) identified a single exception, the *D*_6*h*_-symmetric complex B_7_Be_6_B_7_ (Figure 8).^1^ In this structure, a hexagonal Be_6_ ring is perfectly aligned between two aromatic B_7_ wheels, yielding a closed-shell ^1^A_1g_ system reminiscent of ferrocene. The Be–Be distances (2.10 Å) are consistent with single Be–Be bonds, while the peripheral B–B distances (1.65 Å) are shorter than standard single B–B bonds, reflecting enhanced delocalization.

**Figure 8:**
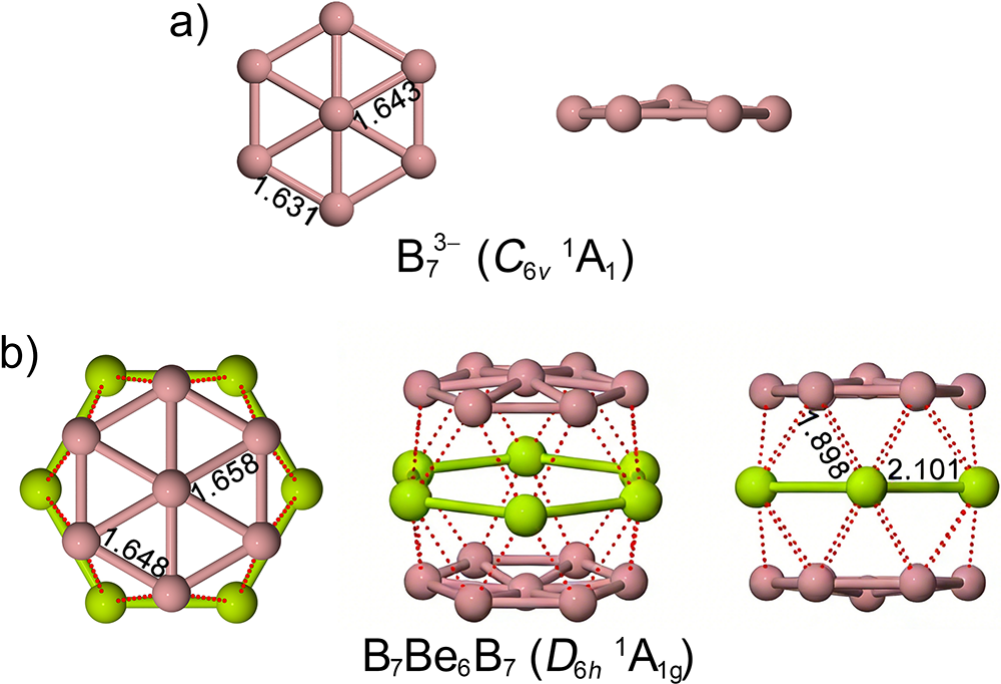
Selected bond distances in Å and different views of a) B_7_^3–^ and b) the global minimum sandwich isomer of B_7_Be_6_B_7_.

CM5 population analysis^90^ shows partial charge transfer from Be to boron, consistent with an idealized [B_7_]^δ−^[Be_6_]^2δ+^[B_7_]^δ−^ charge distribution. Each B_7_ ring closely parallels the aromatic B_7_^3–^ anion, an analogue of the cyclopentadienyl ligand, while preserving both σ and π aromaticity. The Be_6_ ring provides an electronic pathway that couples the two boron decks into a single delocalized framework.

AdNDP analysis further confirms this cooperative bonding pattern (Figure 9). Twelve peripheral 2c–2e σ B–B bonds define the B_7_ units, while six 7c–2e σ and π orbitals extend over both boron rings. In addition, three 20c–2e multicenter bonds connect the Be_6_ layer with the flanking boron wheels. Together, these features describe a dual-aromatic boron–metal sandwich stabilized by a combination of covalent delocalization and electrostatic redistribution, constituting a purely inorganic analogue of classic organometallic sandwiches.

**Figure 9:**
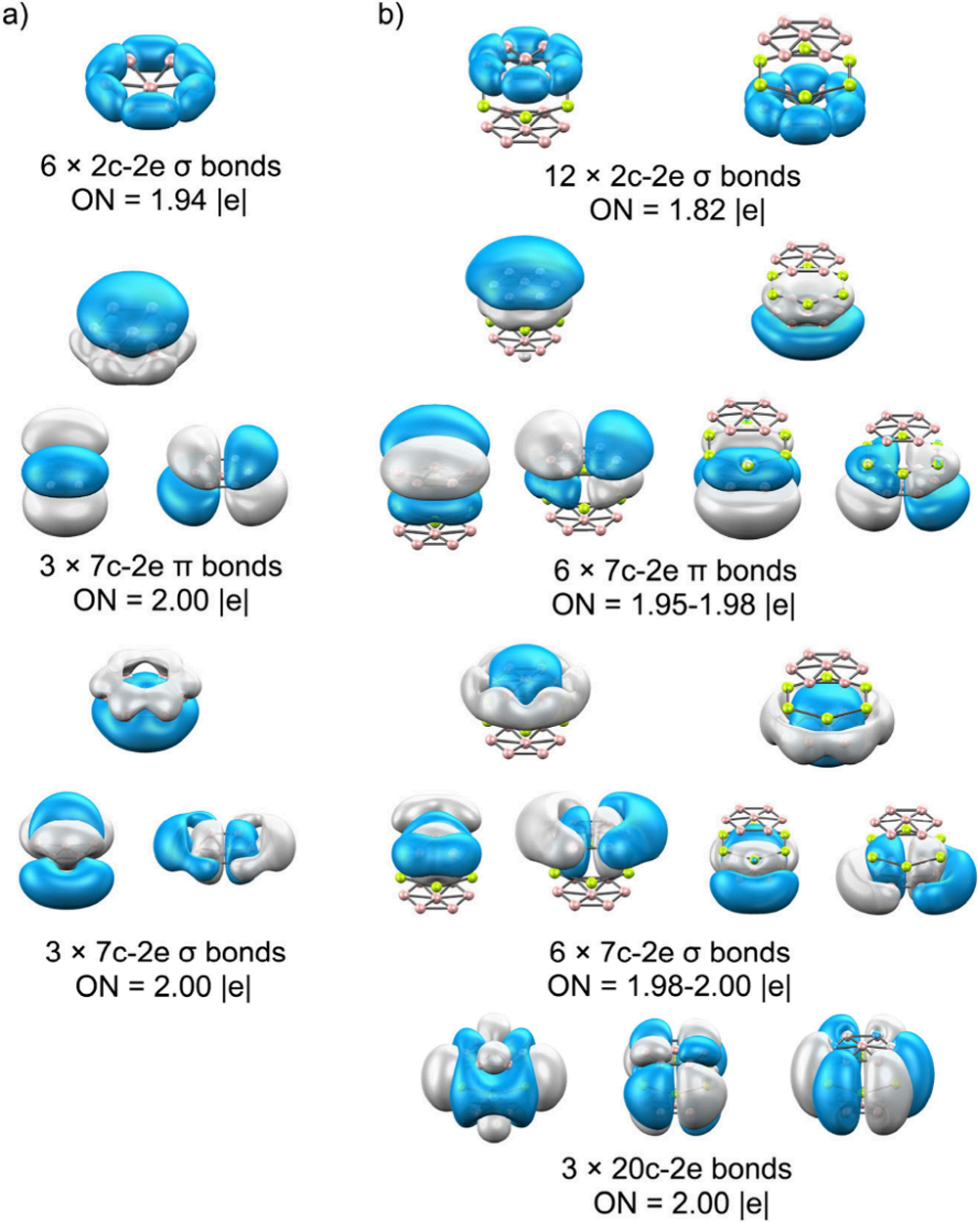
AdNDP analysis of B_7_^3–^ and B_7_Be_6_B_7_. Occupation numbers (ON) are shown in |e|.

### 3.3 A Four-Ring Tubular Boron Motif in Be_2_B_24_^+^

The B_24_ cluster can adopt a variety of structural forms, including quasi-planar sheets, double- and triple-ring tubes, and higher-order tubular motifs, whose relative stability depends sensitively on the charge state.^83–85^ In most cases, the double-ring tubular structure is favored, whereas the planar form becomes preferred for highly reduced species. Three- and four-ring isomers, although conceptually appealing as precursors to extended boron nanotubes, are intrinsically less stable and appear only as high-lying minima in the absence of external stabilization.

Metal doping provides an elegant route to overcome this limitation. A systematic exploration of MB_24_^q^ and M_2_B_24_^q^ clusters (M = alkali or alkaline-earth metal; q = +1, 0, −1) indicates that incorporation of metals stabilizes larger tubular architectures. Among these systems, Be_2_B_24_^+^ is particularly stable, with two Be atoms capping the ends of a four-ring boron tube (Figure 10).^74^ This structure can be viewed as two B_12_ double-ring subunits fused through their central B_6_ rings, with Be atoms positioned axially along the principal axis. The resulting *C*_2*v*_ structure represents the smallest fully closed four-ring tubular boron system in terms of ring count, establishing a structural connection between molecular clusters and extended boron nanotubes.

**Figure 10:**
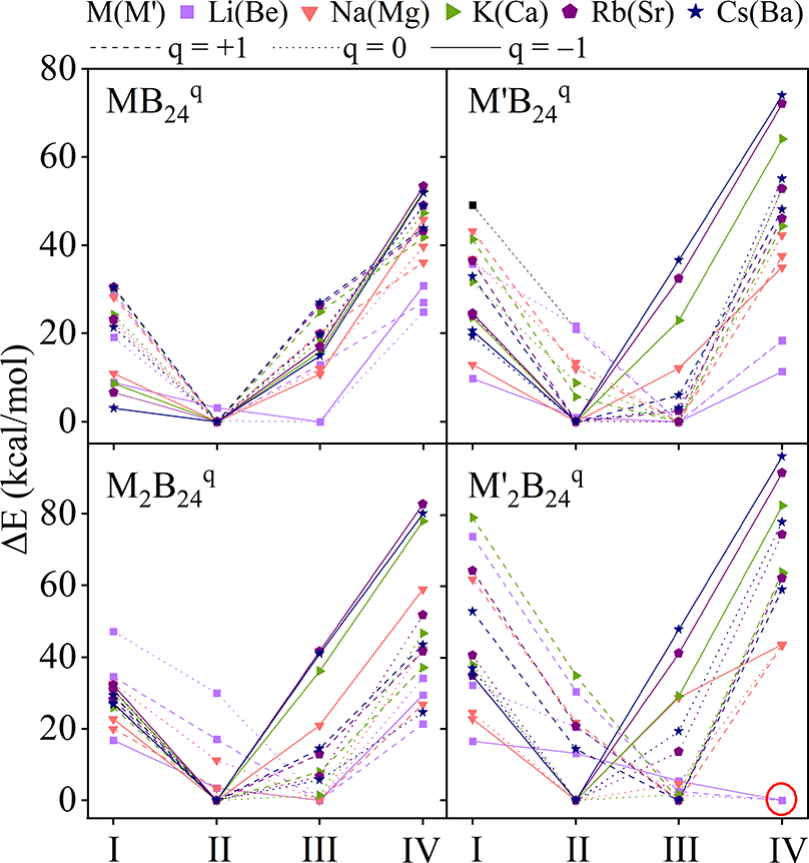
Relative energy (in kcal/mol) of four representative MB_24_^q^ and M_2_B_24_^q^ (M/M′ = alkali/alkaline-earth metals; q = +1, 0, −1) structures, including quasi-planar (**I**), two-ring (**II**), three-ring (**III**) and four-ring (**IV**) tubular boron motifs, computed at the TPSS-D3/def2-TZVP level.

Bonding analysis suggests a cooperative interplay between electrostatic and covalent contributions. Population analyses show charge transfer from Be to the boron framework, on the order of +1.4 to +1.8 |e| per Be, confirming the dominance of the electrostatic component. At the same time, AdNDP reveals an extensive network of multicenter σ bonds delocalized over the entire skeleton, coupling the two B_12_ halves (Figure 11). In addition to localized 2c–2e and 3c–2e B–B bonds, several 26c–2e σ orbitals span the full length of the tube. A singly occupied 24c–1e orbital also contributes to global delocalization, reinforcing the multicenter bonding pattern characteristic of extended boron architectures.

**Figure 11:**
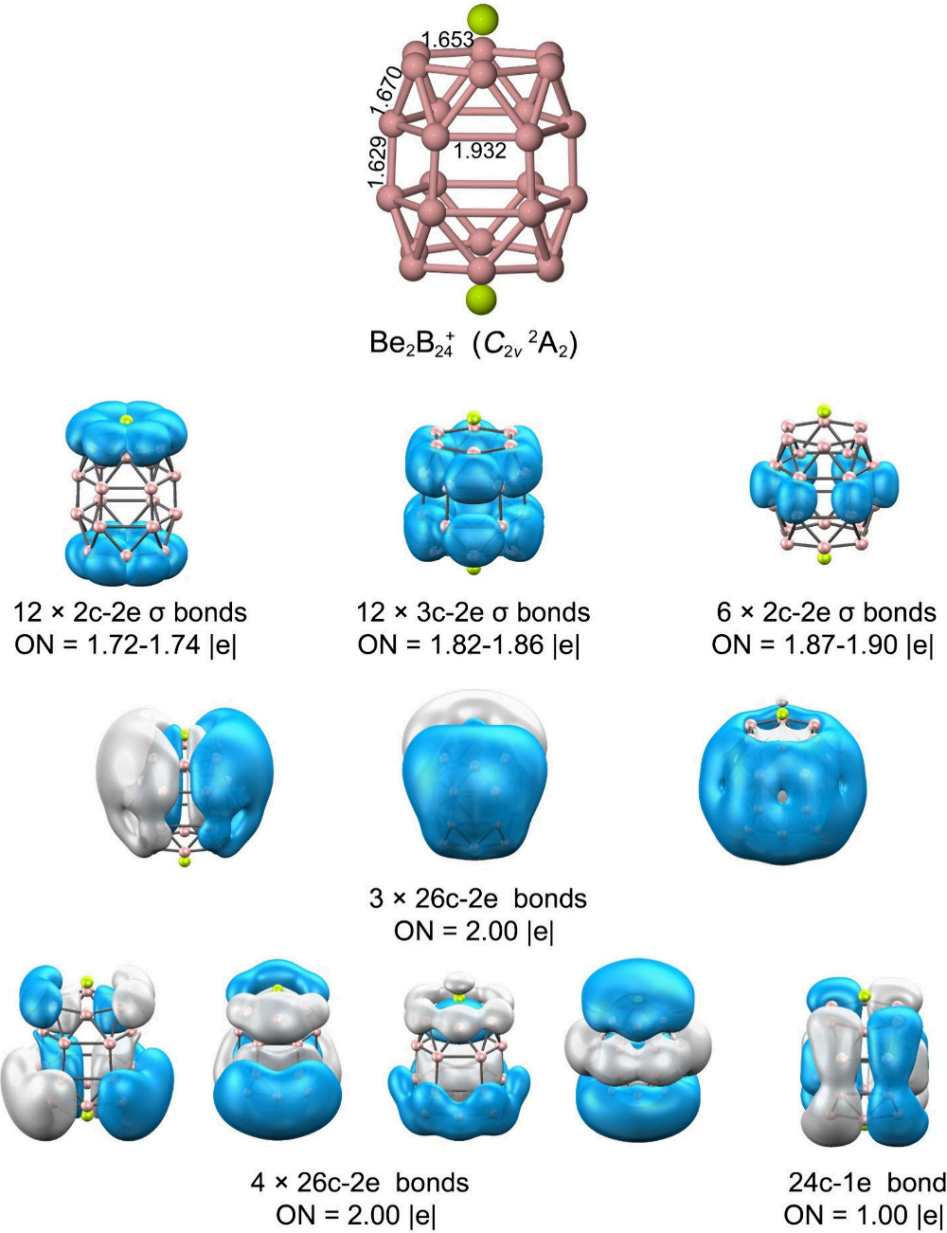
Bond distances (Å) and AdNDP bonding pattern of Be_2_B_24_^+^. Occupation numbers (ON) are given in |e|.

EDA-NOCV provides a quantitative description of these interactions. Fragmentation into a Be_2_^2+^ (1*s* ^4^2*p*_*z*_ ^2^) unit interacting with a quartet B_24_^–^ framework yields an overall attraction composed of roughly 60% covalent and 40% electrostatic contributions. The dominant orbital terms arise from polarized electron-sharing in Be_2_^2+^ *p*_*z*_ ↔ B_24_, complemented by dative interactions, where the Be *s* and *p*_*x*_/*p*_*y*_ orbitals act as electron acceptors. These interactions describe how beryllium, despite being an *s*-block element, mediates both charge redistribution and covalent coupling, enabling the stabilization of extended tubular boron frameworks.

## 4 Covalent Interaction Involving (*n*–1)*d* Orbitals of Heavier Alkaline-Earth Metals and Delocalized Bonds of Boron Clusters

The heavier alkaline-earth elements (Ae = Ca, Sr, Ba) introduce a distinct bonding regime in boron clusters.^4^ Upon descending group 2, their increasing availability of the (n–1)*d* orbitals allows participation in metal–boron covalent interactions, endowing them with a degree of transition-metal character.^91–93^ This behavior departs from the traditional view of alkaline-earth metals as purely electrostatic donors and indicates their capacity to stabilize complex boron frameworks through a combination of electrostatic and covalent contributions.

Computational exploration of Ae_2_B_*x*_ (Ae = Ca, Sr, Ba; x = 8, 18, 30) clusters indicates the participation of the (n–1)*d* orbitals plays a decisive role in determining both symmetry and dimensionality.^4^ At the PBE0-D3/def2-TZVP level,^94,95^ the global minima correspond to symmetric structures, including monocyclic Ae_2_B_8_ rings as well as tubular Ae_2_B_18_, and Ae_2_B_30_ frameworks, each capped by two Ae atoms (Figure 12). Smaller rings exhibit enhanced multiple-bond character, with B–B distances of about 1.55 Å, whereas larger tubes display the typical alternation between short intraring and longer inter-ring bonds. These findings underscore the adaptability of boron’s multicenter bonding to accommodate metal participation while preserving extensive delocalization.

**Figure 12:**
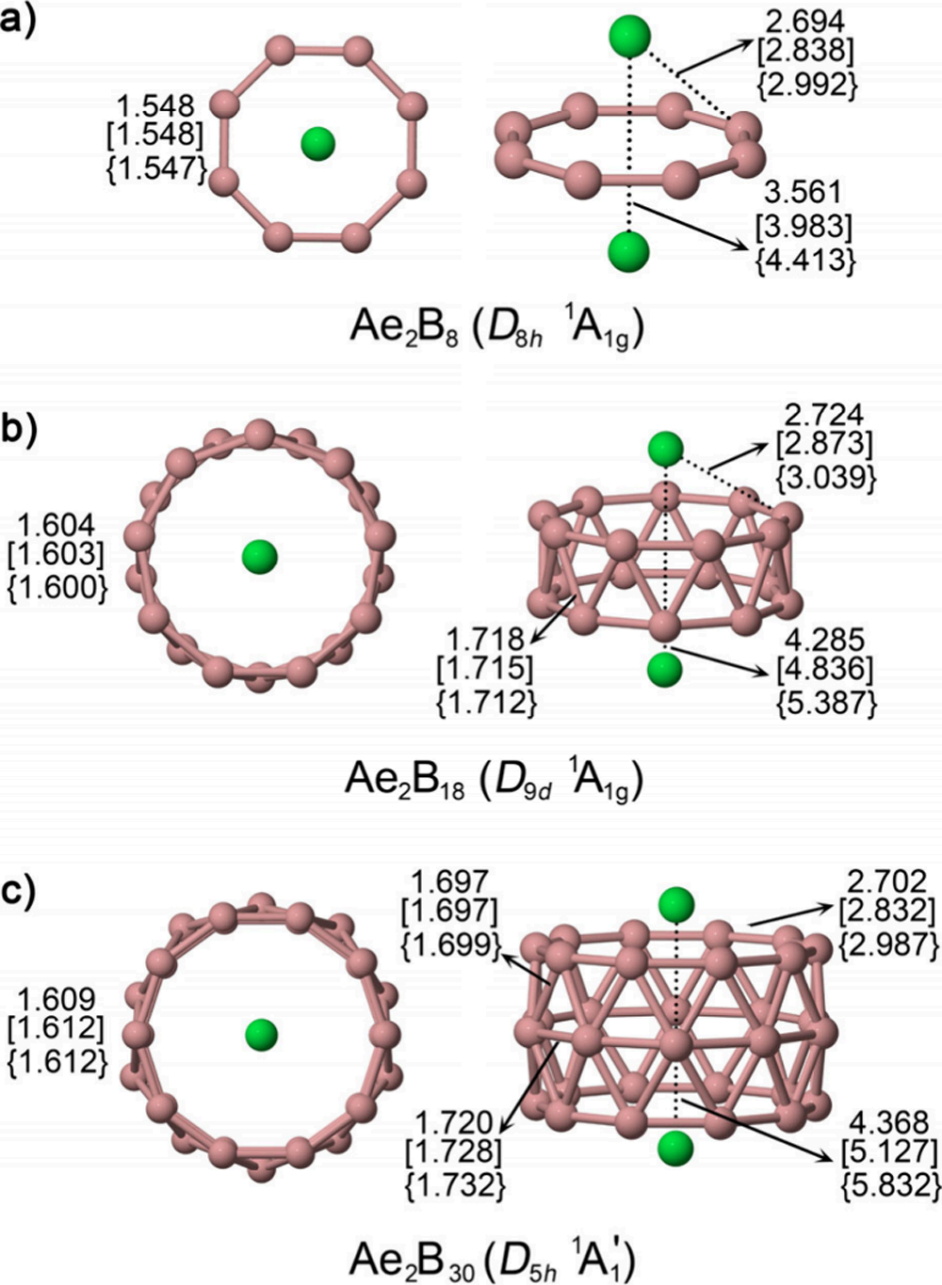
Structures of Ae_2_B_8_, Ae_2_B_18_, and Ae_2_B_30_ (Ae = Ca, [Sr], {Ba}) clusters, shown in top and side views. Bond lengths are given in Å.

Bonding analyses show that Ae_2_B_8_ is characterized by eight localized 2c–2e σ bonds defining the B_8_ ring, together with three delocalized σ and three delocalized π orbitals that confer double aromaticity (Figure 13). In Ae_2_B_18_ and Ae_2_B_30_, delocalization becomes more extensive, spanning multiple B_9_ or B_10_ rings through ten or more multicenter orbitals that couple the boron framework with the Ae centers. In these systems, the heavier Ae atoms contribute through their (n–1)*d* orbitals, which overlap with both σ and π delocalized orbitals of the boron skeleton, giving rise to combined electrostatic and covalent stabilization reminiscent of transition-metal-doped clusters.

**Figure 13:**
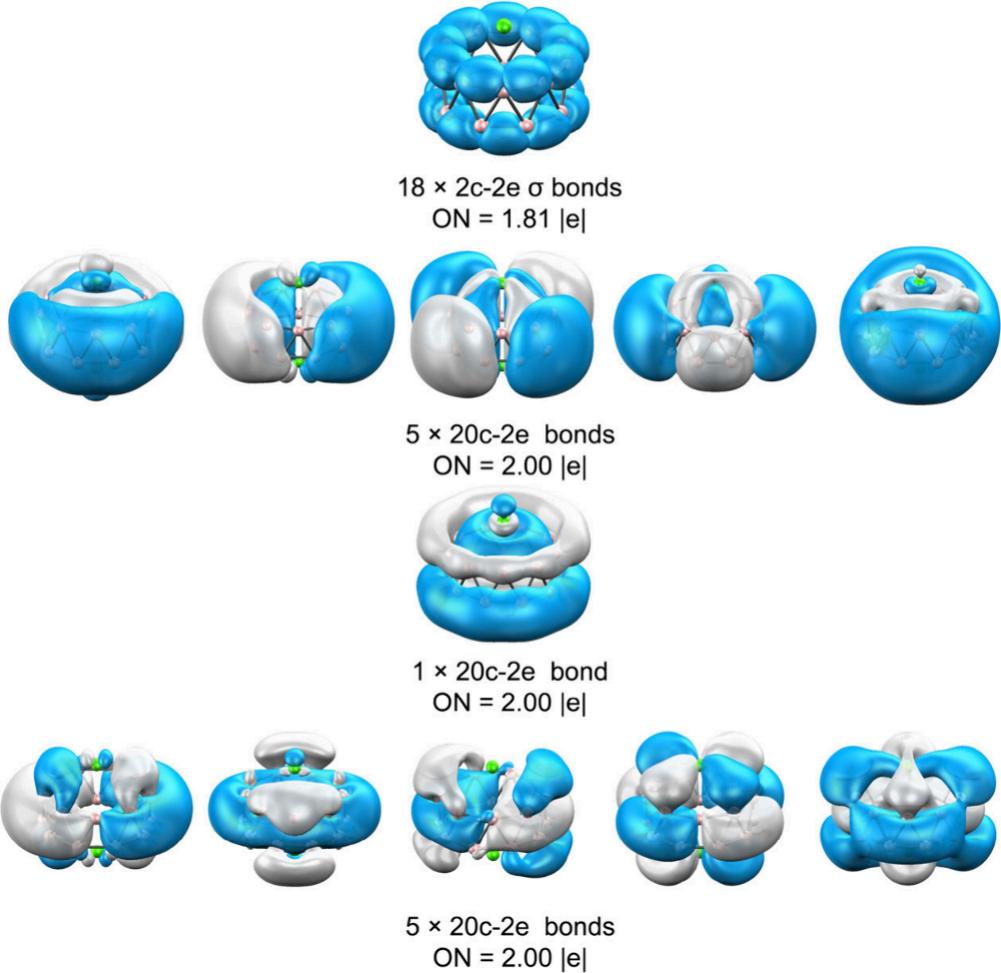
AdNDP bonding analysis of Ae_2_B_18_ (Ae = Ca, Sr, Ba). Occupation numbers (ON) are shown in |e|.

EDA-NOCV provides a quantitative description of these interactions. In Ae_2_B_8_, contributions associated with *d*-orbital participation account for about 60% of the covalent term, increasing to approximately 70–80% in Ae_2_B_18_ and Ae_2_B_30_. This strong *d*-orbital participation is accompanied by substantial charge transfer from Ae to boron, reinforcing the electrostatic component. Accordingly, the bonding can be described as dual in nature, with charge donation providing anchors for the interaction and covalent *d*–(σ,π) overlap contributing to directional bonding and high symmetry.

Collectively, these results show that heavier alkaline-earth elements occupy an intermediate position between classical *s*-block and transition-metal chemistry. Their dual bonding character enables the stabilization of highly symmetric, multicenter-bonded boron frameworks that cannot be achieved through purely electrostatic interactions alone. The progression from lithium-dominated electrostatic control^3,72,73^ through beryllium-based *s*–*p* covalent contributions,^1,2,74^ to the increasing involvement of *d*-orbital in Ca, Sr, and Ba,^4,76^ delineates a continuous spectrum of bonding modes across the alkali and alkaline-earth series. This trend illustrates how systematic changes in electronic structure modulate geometry and bonding in boron–metal systems.

## 5 Summary and Outlook

The chemistry of boron remains a remarkable testing ground for examining bonding, delocalization, and periodic trends. Its intrinsic electron deficiency favors multicenter σ and π interactions that depart from classical two-center bonding and metallic delocalization. Across the alkali and alkaline-earth series, systematic metal doping reveals that electrostatic and covalent contributions evolve continuously and cooperatively, governing the geometry, symmetry, and electronic structure of boron clusters.

Lithium shows how predominantly electrostatic stabilization can induce pronounced structural reorganizations, driving the quasi-planar → tubular → cage evolution in B_12_-based frameworks. In contrast, beryllium introduces a non-negligible covalent component through interactions involving its 2*s* and 2*p* orbitals, which, owing to the small size and favorable energetic matching of Be with the boron framework, stabilizes closed and multilayered motifs such as beryllo-borospherenes, boron–beryllium sandwiches, and multiring tubes. This behavior distinguishes beryllium-doped clusters from lithium-based systems, where stabilization is largely electrostatic, and from heavier alkaline-earth elements (Ca, Sr, Ba), where (n–1)*d* orbitals dominate the covalent contribution. These trends indicate that the nature of bonding is not discrete but continuous, evolving smoothly as a function of charge transfer and orbital participation across the alkali and alkaline-earth series.

These systems highlight that multicenter bonding is not a curiosity of boron but represents a general mechanism for stabilizing electron-deficient systems. They challenge the conventional separation between “main-group” and “transition-metal” chemistry by revealing a continuous spectrum of bonding behaviors where orbital availability, not formal classification, dictates structure. Extending these concepts from finite clusters to extended solids offers an exciting frontier. If analogous electrostatic and covalent mechanisms operate in bulk or 2D materials, boron–metal frameworks may exhibit tunable electronic, magnetic, and catalytic properties. In this context, computational exploration guided by bonding analysis may thus lead to predictive design rules for materials where geometry and electronic function are intertwined. More broadly, these findings encourage a rethinking of chemical periodicity, not as a rigid classification, but as a continuum of bonding possibilities.

From an experimental perspective, the predicted boron–metal clusters should be accessible using established gas-phase techniques, particularly laser ablation in molecular beams combined with mass-selective detection, which is routinely employed for cationic boron clusters.

Ultimately, the study of boron–metal interactions emphasizes that structural beauty in chemistry often arises from conceptual simplicity: when charge donation and orbital sharing cooperate, the boundaries between molecule and material, or between main-group and transition-metal behavior, become fluid. This interplay continues to define boron as one of chemistry’s most versatile and intellectually provocative elements.
